# CT Chest and pulmonary functional changes in patients with HTLV-associated myelopathy in the Eastern Brazilian Amazon

**DOI:** 10.1371/journal.pone.0186055

**Published:** 2017-11-02

**Authors:** Luiz Fábio Magno Falcão, Aline Semblano Carreira Falcão, Rita Catarina Medeiros Sousa, Waldônio de Brito Vieira, Robson Tadachi Moraes de Oliveira, Valéria Marques Ferreira Normando, George Alberto da Silva Dias, Marcio Clementino de Souza Santos, Rodrigo Santiago Barbosa Rocha, Gilberto Toshimitsu Yoshikawa, Roberta Vilela Lopes Koyama, Satomi Fujihara, Víctor Augusto Cavaleiro Corrêa, Hellen Thais Fuzii, Juarez Aantônio Simões Quaresma

**Affiliations:** 1 Center for Biological Sciences and Health, Pará State University, Belém, Pará, Brazil; 2 Division of Infectious Diseases, Evandro Chagas Institute, Ananindeua, Pará, Brazil; 3 Division of Infectious Diseases, Tropical Medicine Center, Federal University of Pará, Belém, Pará, Brazil; 4 Radiology servisse, Maternity of the Holy House, Belém, Pará, Brazil; George Mason University, UNITED STATES

## Abstract

The aim of this study was to compare computed tomography (CT) scans of chest and lung function among patients with Human T-Lymphotropic Virus Type 1 (HTLV) with and without HTLV-1 associated myelopathy/tropical spastic paraparesis (HAM/TSP). In this cross-sectional study performed between January 2013 and June 2016, we included 48 patients with HAM/TSP (19 women and 11 men) and without HAM/TSP (12 women and 6 men). We compared CT findings and lung functions of these groups. Patients who had HAM/TSP had abnormal CT findings (*P* = 0.000), including more frequent bronchiectasis (*P* = 0.049), parenchymal bands (*P* = 0.007), interlobular septal thickening (*P* = 0.035), and pleural thickening (*P* = 0.009). In addition, neither patients with HAM/TSP (9/30; 30%) nor the controls (0/18; 0%) had obstructive or restrictive lung disease (*P* = 0.009). HTLV diagnosis should be considered in all patients with abnormal CT findings in whom no other cause is apparent. It is important to remember that lung disease increases the rates of morbidity and mortality in developing countries.

## Introduction

Human T-lymphotropic virus type 1 (HTLV-1) is a retrovirus that is present worldwide; its prevalence is high in South America [1]. Systemic inflammatory diseases associated with HTLV-1 include HTLV-1-associated myelopathy/tropical spastic paraparesis (HAM/TSP), which is characterized by the activation of specific lymphocytes that cross the blood-brain barrier. This results in the production of chemokines and pro-inflammatory cytokines that affect the central nervous system, causing the typical signs and symptoms of spinal cord injury. In addition, individuals with HAM/TSP exhibit signs of immunological dysfunction that are not present in HTLV carriers. Uveitis and arthritis are also inflammatory manifestations associated with infection with this virus [2].

On the other hand, the association between HTLV-1 infection and pulmonary changes is not clear [3,4]; only a few studies have been published, and none has been conducted yet in the Brazilian Amazon. Also, studies have not clearly defined whether patients with established systemic inflammation (e.g. HAM/TSP) differ from HTLV-1 carriers [5]. Thus, the aim of this study was to compare chest computed tomography (CT) findings and lung function among patients with and without HAM/TSP.

## Methods

### Ethical aspects

The present study was approved by the Ethics Committee of the Instituto Evandro Chagas (Opinion No. 292.251) and complied with the Declaration of Helsinki. All subjects gave written informed consent.

### Study and sample calculation

This was a cross-sectional study performed between January 2013 and June 2016. For calculating the sample size, we considered both the prevalence of abnormal pulmonary findings in 30.1% of patients with HTLV-1 infection [5] and 8.3% from a pilot study of 12 patients. The power of the test was 80%, confidence interval 95%, and alpha level 5%, giving an estimated sample of 28 individuals.

### Subjects

The study included individuals of both sexes who were infected with HTLV-1, with and without HAM/TSP. The serological diagnosis of the infection was performed using enzyme-linked immunosorbent assays and confirmed using polymerase chain reaction (PCR). Patients with HAM/TSP were defined as those with non-remitting progressive spastic paraparesis with a degree of gait impairment high enough to be perceived by the patient, with or without sensory symptoms, and with or without urinary and anal sphincter signs or symptoms. Disorders that can resemble HAM/TSP were excluded [6]. None of our patients had a history of congenital conditions, tuberculosis, exposure to chemical irritants, or childhood infections. There were also no documented or clinical features suggesting a diagnosis of pulmonary changes at or prior to the diagnosis of HTLV-1 infection as per the patient. All of our patients were non-smokers.

**Fig 1:**
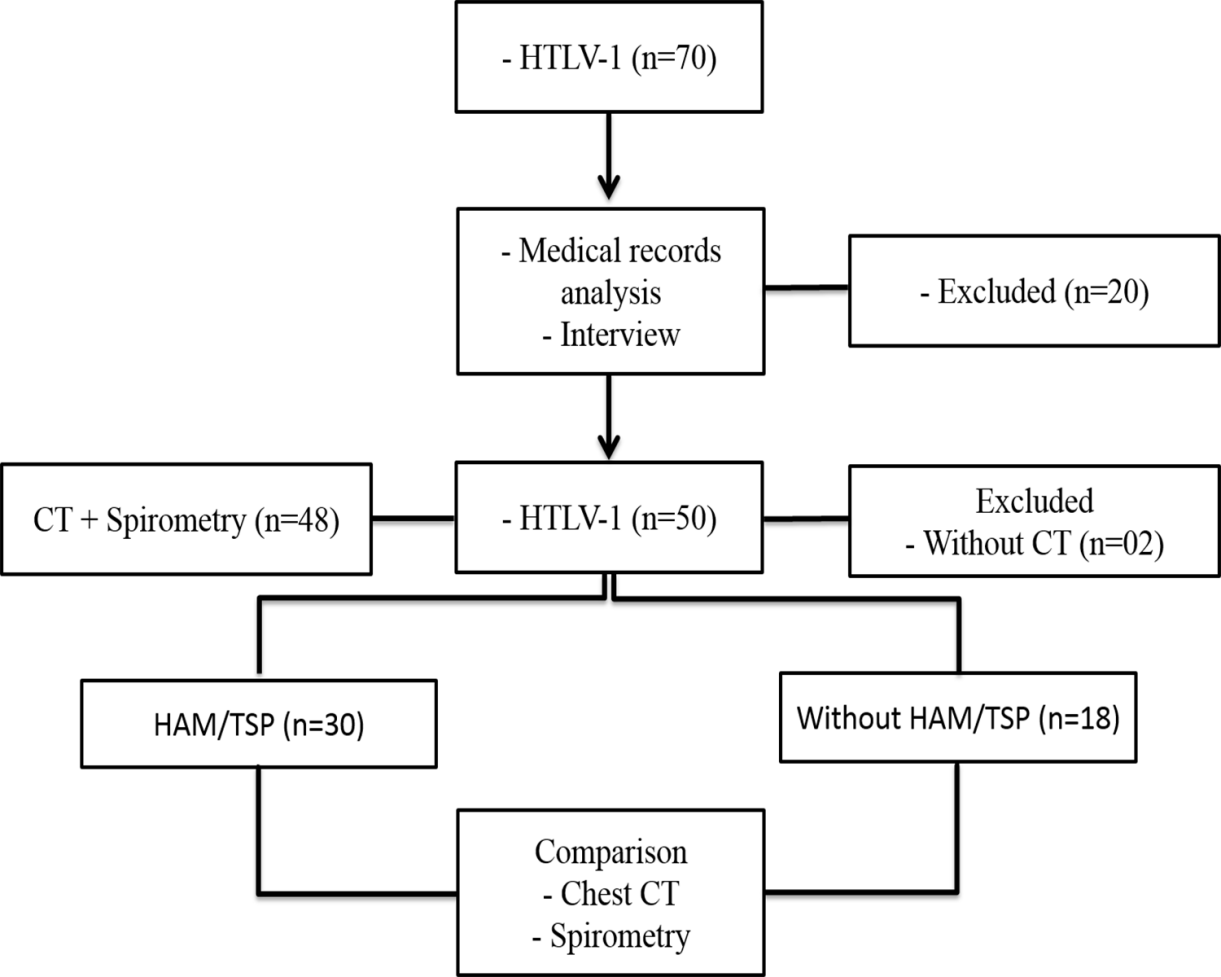
Study flow chart. This flow chart describes the selection of study patients.

Seventy (n = 70) patients were initially selected, but 20 were excluded owing to a history of pulmonary tuberculosis (n = 9), co-infection with human immunodeficiency virus (HIV) (n = 3), haemolytic anaemia (n = 1), scleroderma (n = 1), refusal to participate (n = 1), smoking (n = 2), exposure to dust at work (n = 1), or pregnancy (n = 2). Of the remaining 50 patients, 2 did not undergo a chest CT scan, leaving a total of 48 eligible patients, of which 30 were classified as having HAM/TSP (19 women, aged 25–74 years, mean 52.45±11.8 years, and 11 men, aged 38–64 years, mean 50.36±8.6 years) and 18, as not having HAM/TSP (12 women, aged 39–66, mean 51±9.7 years, and 6 men, aged 47–61 years, mean 52.3±7.5 years) (Fig 1).

### CT scans

Each patient underwent a single examination without medication, sedatives, or intravenous contrast use. In the supine position, each patient took a deep breath followed by momentary apnoea inside the high-resolution GE Multislice BrightSpeed Edge Select CT scanner (GE Healthcare, Chalfont St Giles, UK) to obtain transverse chest images with 1 mm collimation cuts. Each CT scan was analysed by consensus of two radiologists. An Imac computer (Apple Inc., Cupertino, USA) and the OsiriX MD version 5.8.5 software (Pixmeo Company, Bernex, Switzerland) enabled the accurate and comprehensive analysis of pulmonary structures.

The findings of the chest CT images were classified as following: a) bronchiectasis/bronchiolectasis: bronchial dilatation with respect to the accompanying pulmonary artery branch (signet ring sign), lack of tapering of bronchi, and the identification of bronchi within 1 cm of the pleural surface; b) parenchymal bands: an area of scarring in the lung parenchyma, reflecting pleuroparenchymal fibrosis (a parenchymal band is a linear opacity, usually 1–3 mm thick and up to 5 cm long that usually extends to the visceral pleura); c) pleural thickening; d) centrilobular nodules or small nodules with thickening of the wall or filling of the bronchiolar lumen; e) interlobular septal thickening or thickening/scarring of the connective tissue septa separating the secondary pulmonary lobes; f) lung cyst or a rounded area in the lung parenchyma with a well-defined interface; g) ground-glass opacity, defined as increased density of the lung parenchyma that retains visible contours of the vessels and bronchi inside the affected area; and h) a mosaic attenuation pattern or the appearance of regions with distinct attenuation [7,8]. All slices above the level of the diaphragm were evaluated.

### Pulmonary function

We used a Spirolab spirometer^™^ (Medical International Research, WI, USA) to assess pulmonary function, following the guidelines established by the Brazilian Consensus on Spirometry and Brazilian Society of Pulmonology and Phthisiology for the interpretation of the results [9]. We analysed the vital capacity (VC), forced vital capacity (FVC), forced expiratory volume in one second (FEV_1_), the ratio of forced expiratory volume in one second to forced vital capacity (FEV_1_/CVF), the 25%–75% forced expiratory flow (FEF_25–75%_), the ratio of the 25%–75% forced expiratory flow to the forced vital capacity (FEF_25–75%_/FVC), the 25%–75% forced expiratory time (FET_25%–75%_), the maximum forced expiratory flow (FEF_max_), the 50% forced expiratory flow (FEF_50%_), the 75% forced expiratory flow (FEF_75%_), and the maximum voluntary ventilation (MVV). Analysis of the result of each variable was compared between the study groups taking into account obtained and predicted values for each patient in relation to sex.

### Statistical analysis

The data were analysed by using GraphPad Prism version 5.0 (Graphpad Software, Inc., San Diego, CA, USA). The Shapiro–Wilk test was used to evaluate normally distributed variables. Student’s t-test was used in the treatment of normally distributed variables; and Fisher’s exact test, Mann–Whitney, and Wilcoxon tests were used for variables that did not have a normal distribution. An α-level of 0.05 was used to test for statistical significance.

## Results

### CT scans

**Fig 2:**
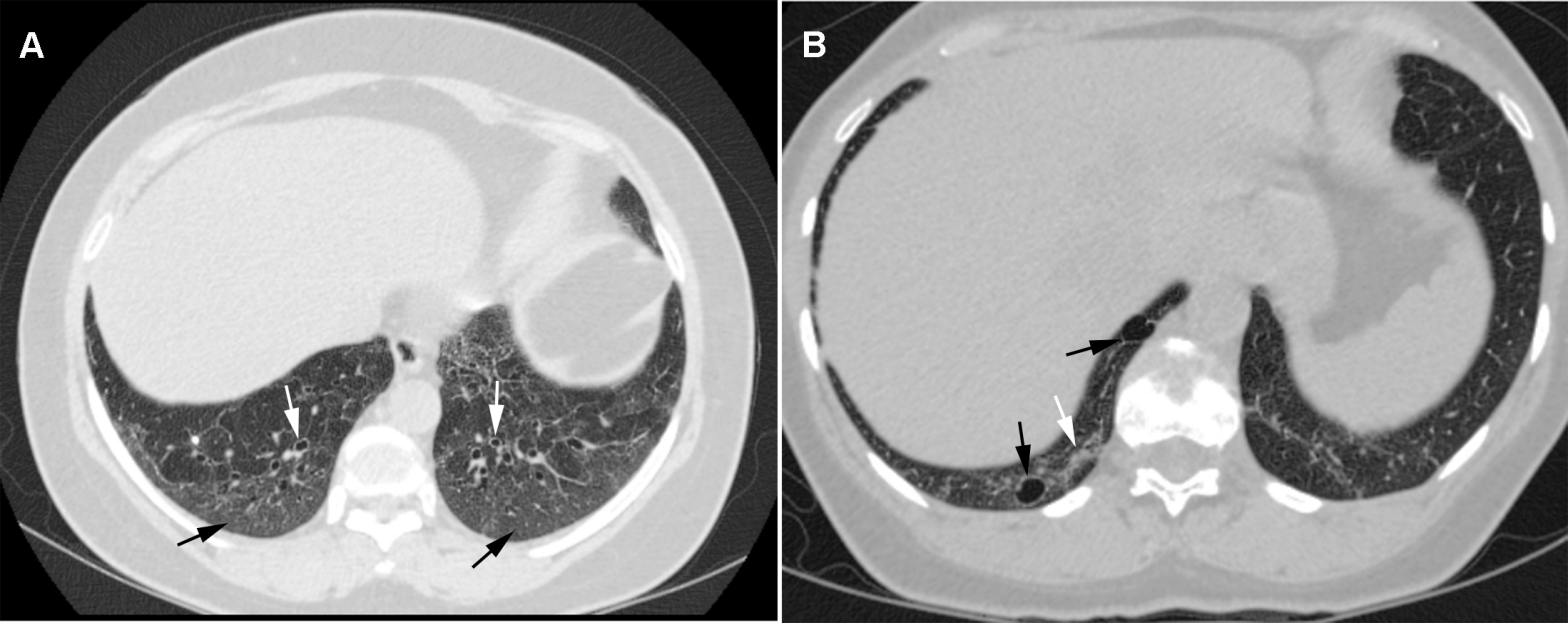
Transverse CT scans obtained from two women aged 60 and 47 years, respectively, suffering from HAM/TSP. a) Bronchiectasis signet ring appearance (white arrows) and ground-glass opacity (black arrows) b) ground-glass opacity (white arrow) and a pulmonary cyst in the right lower lobe (black arrows).

**Fig 3:**
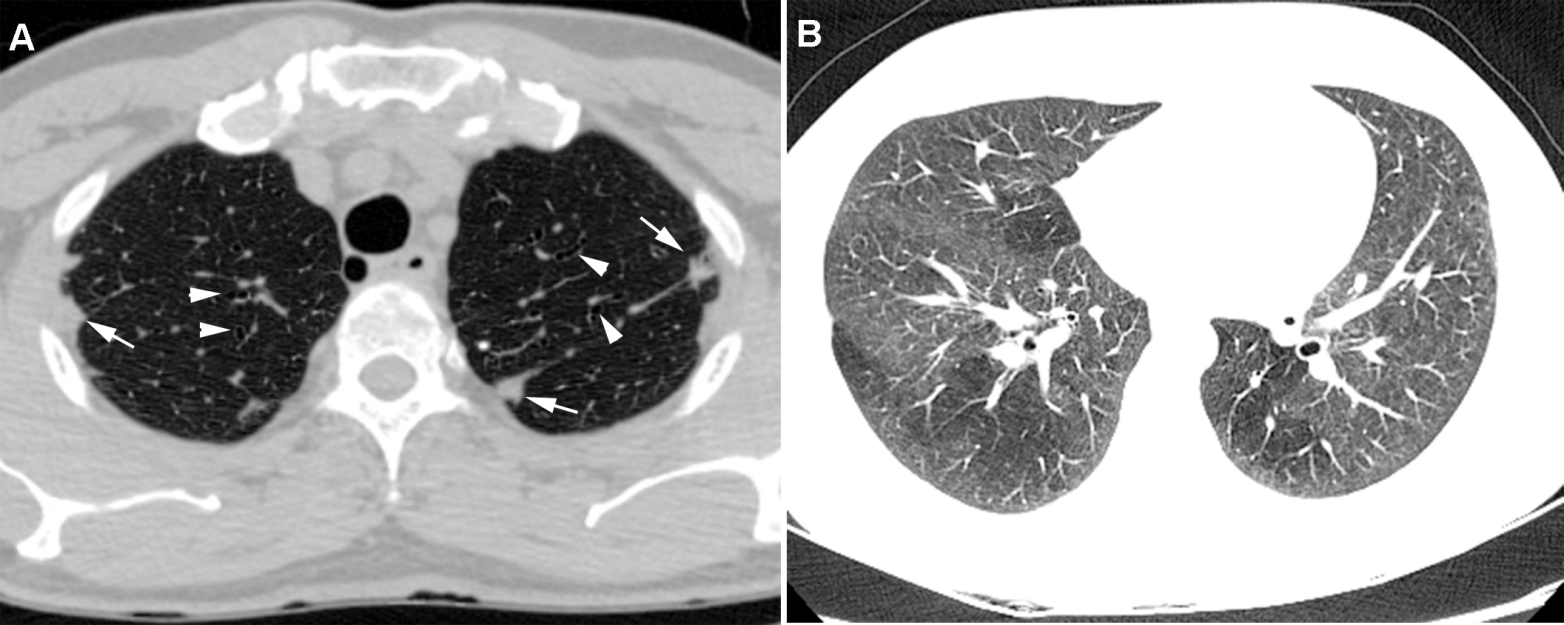
Transverse CT scans of a 59-year-old man and a 25-year-old woman with HAM/TSP. a) Pleural thickening (white arrows) and bronchiectasis (arrowheads), bilaterally b) bilateral mosaic attenuation.

Abnormal CT scan findings were compared between patients with and without HAM/TSP (Figs 2 and 3; Table 1). Some combinations of abnormal CT findings were seen more frequently in patients who had HAM/TSP, such as bronchiectasis and pleural thickening (6/19, 31,5%), bronchiectasis and parenchymal bands (5/19; 26,5%), bronchiectasis and interlobular septal thickening (4/19; 21%), bronchiectasis and centrilobular nodules (4/19; 21%), and centrilobular nodules and parenchymal bands (4/19; 21,1%). Moreover, 37% of the patients (7/19) had a combination of three or more abnormal findings. None of the patients had a history of infectious diseases or pulmonary, occupational, or congenital conditions; moreover, none had been exposed to chemical irritants.

**Table 1:** Abnormal chest CT findings in patients with HAM/TSP and without HAM/TSP.

Variables	HTLV-1		Total	P-value
	HAM/TSP	Without HAM/TSP		
Individuals with abnormal findings	22 (73.5%)	04 (22.0%)	26 (54.0%)	0.000*
Bronchiectasis/bronchiolectasis	12 (40.0%)	02 (11.0%)	14 (29.0%)	0.049*
Parenchymal bands	13 (43.5%)	01 (05.5%)	14 (29.0%)	0.007*
Interlobular septal thickening	07 (23.5%)	00 (00.0%)	07 (14.5%)	0.035*
Pleural thickening	09 (30.0%)	00 (00.0%)	09 (19.0%)	0.009*
Lung cyst	05 (16.5%)	00 (00.0%)	05 (10.5%)	0.141
Centrilobular nodules	06 (20.0%)	01 (05.5%)	07 (14.5%)	0.231
Ground-glass opacity	03 (10.0%)	02 (11.0%)	05 (10.5%)	0.995
Mosaic attenuation	03 (10.0%)	00 (00.0%)	03 (06.3%)	0.281

Fisher's exact test (p<0.05*).

CT: Computed tomography. HTLV-1: Human T lymphotrophic virus type 1. HAM/TSP: HTLV-1-associated myelopathy/tropical spastic paraparesis.

### Pulmonary function

A total of 18,75% (9/48) of patients were diagnosed with obstructive lung disease (OLD) or restrictive lung disease (RLD). Of these, 30% (9/30) had HAM/TSP (4 OLD and 5 RLD), and no patient (0/18) without HAM/TSP had RLD or OLD (*P* = 0.009). The values obtained for each pulmonary function variable were compared with the predicted values in patients with HAM/TSP (Table 2) and without HAM/TSP (Table 3) as well as between the groups (Table 4).

**Table 2:** Volumes, capacities and flows obtained and predicted for women and men with HAM/TSP.

	Women		P-value	Men		P-value
Variables	Obtained	Predicted		Obtained	Predicted	
VC (L)	2.44 ± 0.46	2.95 ± 0.38	0.00*	3.44 ± 0.73	4.23 ± 0.26	0.01*
FVC (L)	2.53 ± 0.42	2.95 ± 0.38	0.00*	3.48 ± 0.74	4.23 ± 0.26	0.02*
FEV_1_ (L)	2.00 ± 0.43	2.42 ± 0.31	0.00*	2.80 ± 0.56	3.47 ± 0.25	0.00*
FEV_1_/CVF (%)	78.8 ± 8.99	81.6 ± 1.84	0.87	81.07 ± 7.8	81.73 ± 2.13	0.81
FEF_25-75_ (L/min)	1.99 ± 0.79	2.37 ± 0.29	0.12	2.74 ± 0.84	3.50 ± 0.46	0.01*
FET_25-75_ (s)	0.79 ± 0.61	0.58 ± 0.05	0.48	0.67 ± 0.20	0.60 ± 0.05	0.27
FEF_máx_ (L/s)	4.64 ± 1.26	6.74 ± 0.50	0.00*	6.68 ± 1.96	10.54 ± 0.29	0.00*
FEF_50%_ (L/s)	2.68 ± 1.13	3.08 ± 0.28	0.23	3.44 ± 0.86	4.43 ± 0.44	0.00*
FEF_75%_ (L/s)	0.83 ± 0.30	0.87 ± 0.17	0.67	1.26 ± 0.81	1.34 ± 0.27	0.76
MVV (L/min)	71.5 ± 14.38	90.27 ± 11.99	0.00*	97.23 ± 20	129.7 ± 10.1	0.00*

Data are expressed as mean±standard deviation.

Paired Student's t test (p<0.05*).

Wilcoxon test (p<0.05*).

VC: vital capacity. FVC: forced vital capacity. FEV_1_: forced expiratory volume in one second. FEV_1_/CVF: ratio of forced expiratory volume in one second to forced vital capacity. FEF_25-75%_: 25%–75% forced expiratory flow. FEF_25-75%_/FVC: ratio of the 25%–75% forced expiratory flow and the forced vital capacity. FET_25%-75%_: 25%–75% forced expiratory time. FEF_máx_: maximum forced expiratory flow. FEF_50%_: 505 forced expiratory flow. FEF_75%_: 75% forced expiratory flow. MMV: maximum voluntary ventilation. HAM/TSP: HTLV-1-associated myelopathy/tropical spastic paraparesis. L: liters. min: minute. s: second.

**Table 3:** Volumes, capacities and flows obtained and predicted for women and men without HAM/TSP.

	Women		P-value	Men		P-value
Variables	Obtained	Predicted		Obtained	Predicted	
VC (L)	2.69 ± 0.32	2.83 ± 032	0.10	4.26 ± 0.64	4.32 ± 0.31	0.88
FVC (L)	2.77 ± 0.40	2.83 ± 0.32	0.56	4.76 ± 0.76	4.32 ± 0.31	0.27
FEV_1_ (L)	2.34 ± 0.35	2.34 ± 0.30	0.92	3.81 ± 0.56	3.49 ± 0.21	0.34
FEV_1_/CVF (%)	84.26 ± 4.53	82.49 ± 1.88	0.20	79.74 ± 5.33	80.76 ± 2.07	0.71
FEF_25-75_ (L/min)	2.79 ± 0.76	2.4 ± 0.39	0.09	3.64 ± 0.76	3.34 ± 0.28	0.53
FET_25-75_ (s)	0.52 ± 0.13	0.57 ± 0.06	0.22	0.66 ± 0.13	0.61 ± 0.03	0.54
FEF_máx_ (L/s)	5.89 ± 1.22	6.55 ± 0.36	0.03*	8.65 ± 0.63	10.6 ± 0.31	0.00*
FEF_50%_ (L/s)	3.53 ± 0.9	3.10 ± 0.37	0.10	4.63 ± 1.1	4.28 ± 0.27	0.56
FEF_75%_ (L/s)	1.10 ± 0.39	0.90 ± 0.24	0.12	1.49 ± 0.49	1.24 ± 0.16	0.44
MVV (L/min)	85.66 ± 15.25	87.65 ± 27.26	0.73	139.9 ± 22	129.3 ± 8.06	0.40

Data are expressed as mean±standard deviation.

Paired Student's t test (p<0.05*).

Wilcoxon test (p<0.05*).

VC: vital capacity. FVC: forced vital capacity. FEV_1_: forced expiratory volume in one second. FEV_1_/CVF: ratio of forced expiratory volume in one second to forced vital capacity. FEF_25-75%_: 25%–75% forced expiratory flow. FEF_25-75%_/FVC: ratio of the 25%–75% forced expiratory flow and the forced vital capacity. FET_25%-75%_: 25%–75% forced expiratory time. FEF_máx_: maximum forced expiratory flow. FEF_50%_: 505 forced expiratory flow. FEF_75%_: 75% forced expiratory flow. MMV: maximum voluntary ventilation. HAM/TSP: HTLV-1-associated myelopathy/tropical spastic paraparesis. L: liters. min: minute. s: second.

**Table 4:** Volumes, capacities and flows of women and men with HAM/TSP and without HAM/TSP.

	HTLV-1-women		p-value	HTLV-1-men		p-value
Variables	HAM/TSP	Without HAM/TSP		HAM/TSP	Without HAM/TSP	
VC (L)	2.44 ± 0.21	2.69 ± 0.10	0.11	3.44 ± 0.73	4.26 ± 0.64	0.08
FVC (L)	2.53 ± 0.17	2.77 ± 0.16	0.14	3.48 ± 0.74	4.7 ± 0.76	0.01*
FEV_1_ (L)	2.00 ± 0.19	2.34 ± 0.12	0.03*	2.80 ± 0.56	3.81 ± 0.56	0.01*
FEV_1_/CVF (%)	78.82 ± 8.99	84.26 ± 4.53	0.03*	81.07 ± 781	79.74 ± 5.33	0.76
FEF_25-75_ (L/min)	1.99 ± 0.79	2.79 ± 0.76	0.01*	2.74 ± 0.84	3.64 ± 0.76	0.09
FEF_25-75_/FVC (%)	0.76 ± 0.25	1.00 ± 0.24	0.02*	0.80 ± 0.29	0.76 ± 0.14	0.80
FET_25-75_ (s)	0.79 ± 0.61	0.52 ± 0.13	0.04*	0.67 ± 0.20	0.66 ± 0.13	0.93
FEF_máx_ (L/s)	4.64 ± 1.26	5.89 ± 1.22	0.01*	6.68 ± 1.96	8.65 ± 0.63	0.08
FEF_50%_ (L/s)	2.68 ± 1.13	3.53 ± 0.9	0.02*	3.44 ± 0.86	4.63 ± 1.10	0.05*
FEF_75%_ (L/s)	0.83 ± 0.30	1.1 ± 0.39	0.06	1.26 ± 0.81	1.49 ± 0.49	0.61
MVV (L/min)	71.5 ± 14.38	85.6 ± 15.2	0.02*	97.2 ± 20.0	139.9 ± 22.0	0.00*

Data are expressed as mean±standard deviation.

Student's t test (p<0.05*).

Mann-Whitney test (p<0.05*).

VC: vital capacity. FVC: forced vital capacity. FEV_1_: forced expiratory volume in one second. FEV_1_/CVF: ratio of forced expiratory volume in one second to forced vital capacity. FEF_25-75%_: 25%–75% forced expiratory flow. FEF_25-75%_/FVC: ratio of the 25%–75% forced expiratory flow and the forced vital capacity. FET_25%-75%_: 25%–75% forced expiratory time. FEF_máx_: maximum forced expiratory flow. FEF_50%_: 505 forced expiratory flow. FEF_75%_: 75% forced expiratory flow. MMV: maximum voluntary ventilation. HAM/TSP: HTLV-1-associated myelopathy/tropical spastic paraparesis. L: liters. min: minute. s: second.

## Clinical findings

Dyspnoea was present in 33.5% (16/48) of all study patients, of whom, 46.5% (14/30) had HAM/TSP and 11% (2/18) did not have HAM/TSP (p = 0.013). Hypersecretion was observed in 20% (6/30) of the patients in the HAM/TSP group and none (0/18) without HAM/TSP (p = 0.07). The combination of the two symptoms was present in 10.5% (4/30) of patients with HAM/TSP and 0% (0/18) without HAM/TSP (p = 0.156). Cough has not been reported as a current symptom.

## Discussion

The most comprehensive report of pulmonary imaging findings in patients with HTLV infection to date is a retrospective study involving 320 asymptomatic individuals with abnormal CT findings in 30.1% of the patients [5]. However, pulmonary involvement in HAM/TSP was first described in 1987 [2]. Since then, laboratory and radiological evidence have been demonstrated [2, 4, 10,11,12], although radiological and pulmonary function analyses involving patients with HAM/TSP, as done in this study, have not been previously performed. In addition, no study has been conducted among individuals from the Brazilian Amazon region.

Another study involving 72 indigenous Australians concluded that HTLV-1 infection was predictive of bronchiectasis (*P* = 0.006) and death (*P* = 0.004) resulting from inflammation mediated by the virus [11]. Other studies have reported similar results [12, 13]. We believe that abnormal dilation of the bronchi or bronchioles is more frequently found in patients with HAM/TSP than that in the general population. It is possible that the high prevalence of bronchiectasis is linked to HTLV-1-associated inflammatory disease [3]. A history of cystic fibrosis, pneumonia, exposure to toxic dust, viral infections, or lung disease in childhood, which are common causes of bronchiectasis, were not reported by the patients in our study.

Areas of fibrosis were frequently seen in the CT images, including traction bronchiectasis caused by the effect of elastic recoil exerted on the intrapulmonary airways, parenchymal bands, interlobular septal thickening, and pleural thickening. A study conducted in 2003 investigated the presence of cryptogenic fibrosing alveolitis (chronic interstitial lung disease of unknown cause characterized by inflammation and fibrosis of the lung parenchyma) in 72 patients with and without HTLV-1 infection. The results showed that the prevalence of cryptogenic fibrosing alveolitis in individuals with HTLV-1 infection was 25%; of these patients, 89% had HAM/TSP (*P*<0.05) [14]. It is possible that the high prevalence of fibrosis is linked to the fact that the viral protein p40^*tax*^ induces the production of matrix metalloproteinase-2 and interferon gamma-induced protein 10 in the lungs [14,15].

There are few studies on lung function in patients with HTLV-1 infection. Patients with HTLV-1 infection had lower VC and FEV_1_ values than healthy subjects [14,16]. Different findings have been published [17]. The low VC and/or FVC values are owing to RLD. Furthermore, FEV_1_, when reduced, is associated with airway obstruction. A reduction in the peak flow is very sensitive in most diseases that affect the lungs. FEF_50%_ is altered in the early stages of obstructive lung disease. The reduction in FEF_25–75%_ is linked to histological changes in the peripheral airways and obstruction. Finally, changes in MVV may be present both in diseases that affect the lungs as well as in adverse conditions that alter the mobility of the rib cage [9]. It is possible that the abnormal CT findings, including airway and lung scarring lesions, and the low mobility that affects patients with HAM/TSP (many of them use running auxiliary devices) play a key role in pulmonary function changes.

Many studies point to the importance of local immune and inflammatory responses in the development of injuries that result in tissue damage [2, 4, 10]. A limitation of this study was that we did not analyse the presence of inflammatory mediators in the bronchoalveolar lavage fluid of the study patients. Future studies could examine the presence of such mediators in bronchoalveolar lavage fluid using groups of patients similar to those in this study.

In conclusion, abnormal CT findings are seen in patients with HTLV-1 infection, especially those who develop HAM/TSP. Moreover, pulmonary involvement impacts pulmonary function. Our results emphasise the importance of screening patients with HAM/TSP for pulmonary disease and that CT imaging should be requested in these patients. Furthermore, HTLV diagnosis should be considered in all patients with abnormal CT findings in whom no other cause is apparent. It is important to remember that lung disease increases the rates of morbidity and mortality in developing countries.

## Supporting information

S1 Fig Lung cyst. (TIF)

S2 Fig Pleural thickening. (TIF)

S3 Fig Interlobular septal thickening or thickening/scarring. (TIF)

S4 Fig Centrilobular nodules. (TIF)

S1 Table Abbreviation list. (DOCX)
