# Correction: CT Chest and pulmonary functional changes in patients with HTLV-associated myelopathy in the Eastern Brazilian Amazon

**DOI:** 10.1371/journal.pone.0190436

**Published:** 2017-12-27

**Authors:** Luiz Fábio Magno Falcão, Aline Semblano Carreira Falcão, Rita Catarina Medeiros Sousa, Waldônio de Brito Vieira, Robson Tadachi Moraes de Oliveira, Valéria Marques Ferreira Normando, George Alberto da Silva Dias, Marcio Clementino de Souza Santos, Rodrigo Santiago Barbosa Rocha, Gilberto Toshimitsu Yoshikawa, Roberta Vilela Lopes Koyama, Satomi Fujihara, Víctor Augusto Cavaleiro Corrêa, Hellen Thais Fuzii, Juarez Antônio Simões Quaresma

The last author’s name is spelled incorrectly. The correct name is: Juarez Antônio Simões Quaresma.
