# Supplementary figures and images for: CT Chest and pulmonary functional changes in patients with HTLV-associated myelopathy in the Eastern Brazilian Amazon
Source: PLoS One. 2017 Nov 2;12(11):e0186055. doi: 10.1371/journal.pone.0186055 (PMC5667869; doi:10.1371/journal.pone.0186055)

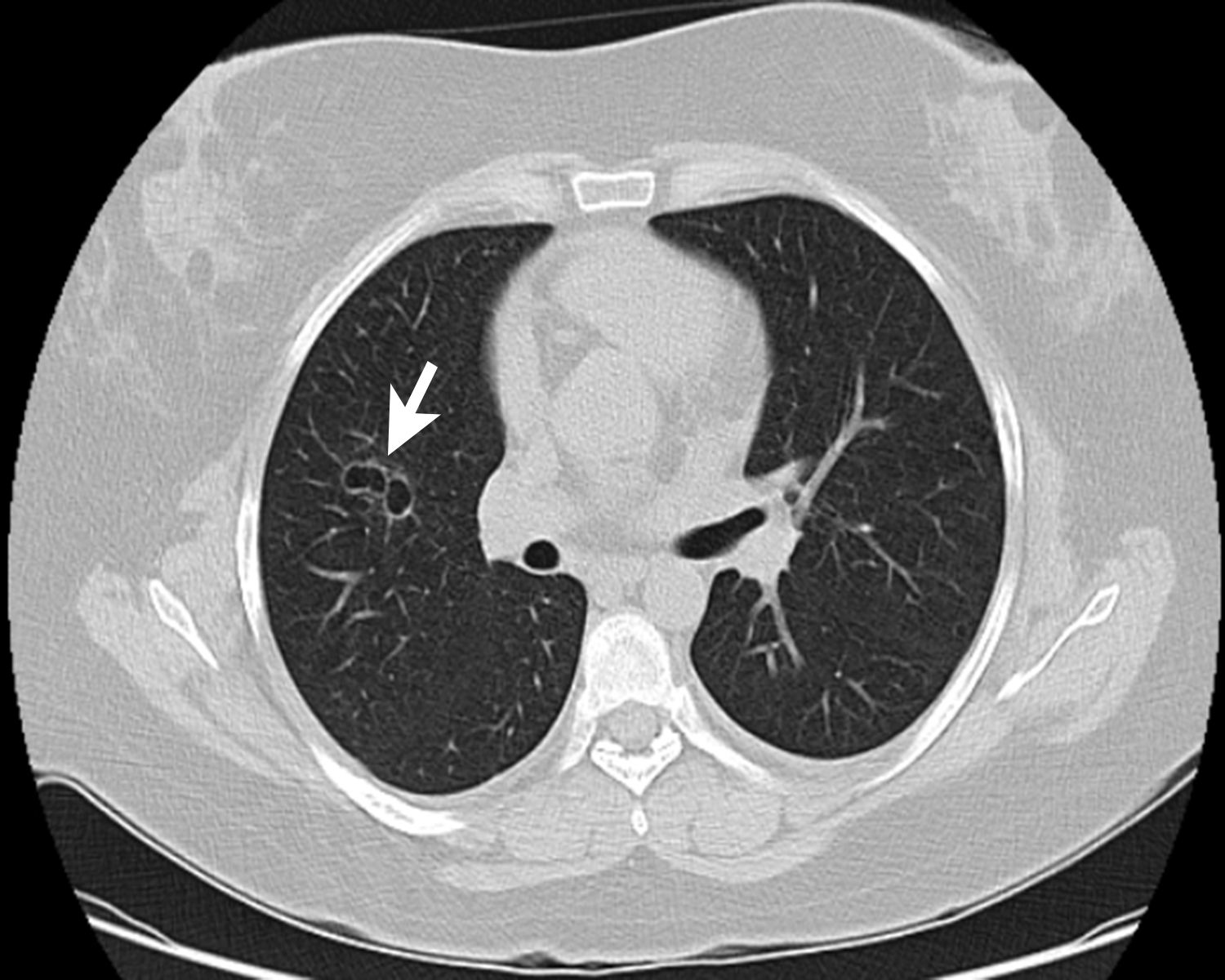

Supplement: S1 Fig — (TIF) [file pone.0186055.s001.tif]

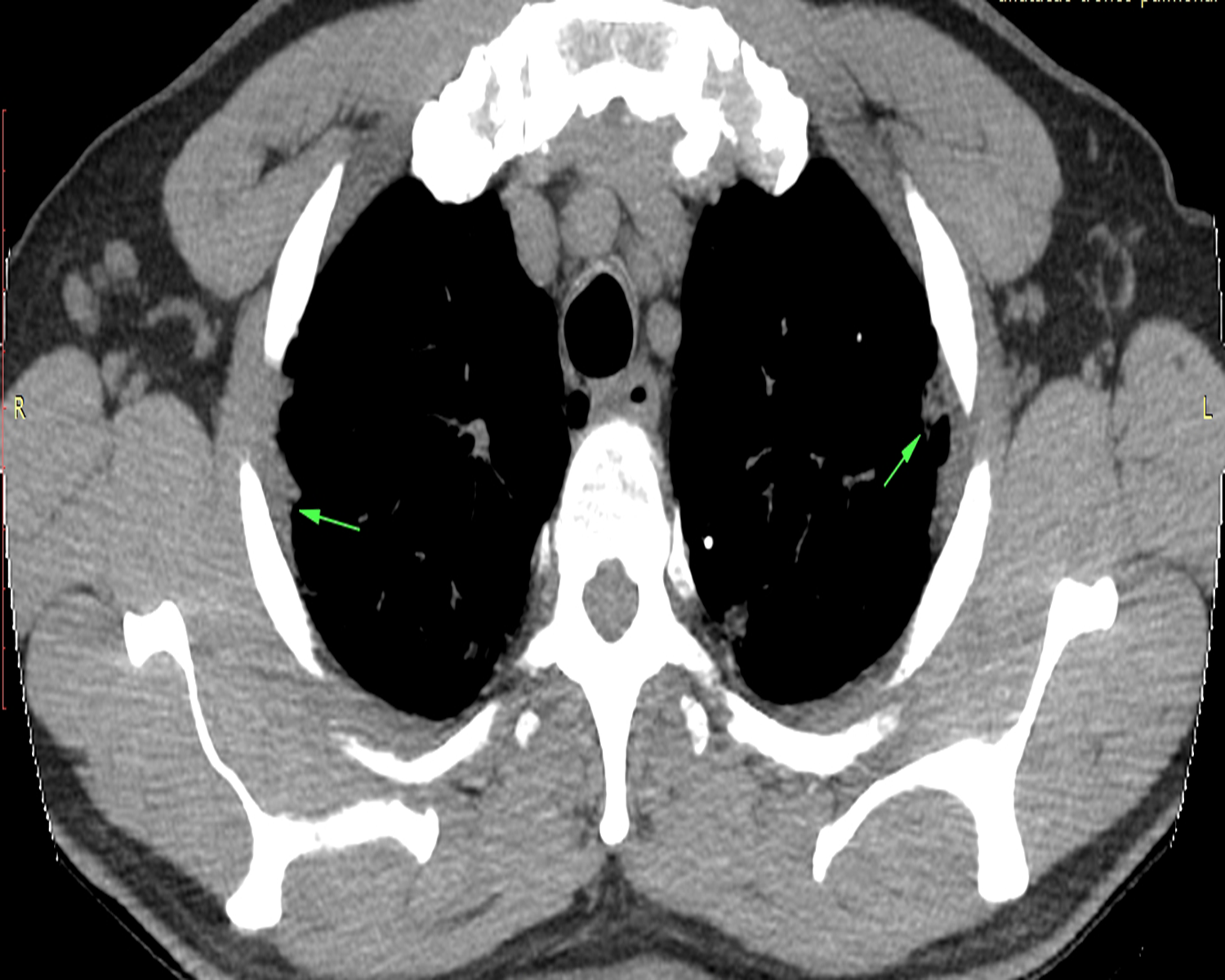

Supplement: S2 Fig — (TIF) [file pone.0186055.s002.tif]

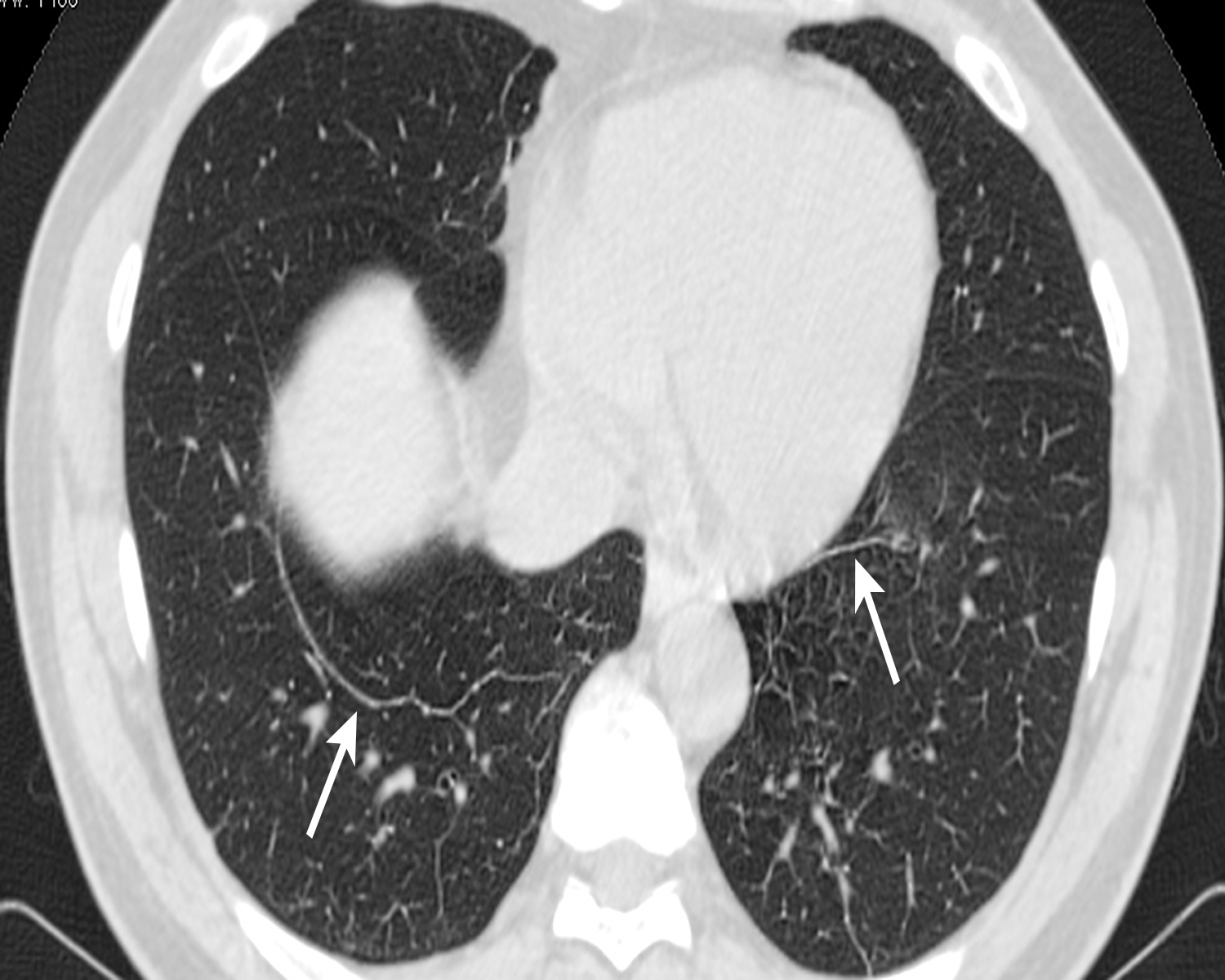

Supplement: S3 Fig — (TIF) [file pone.0186055.s003.tif]

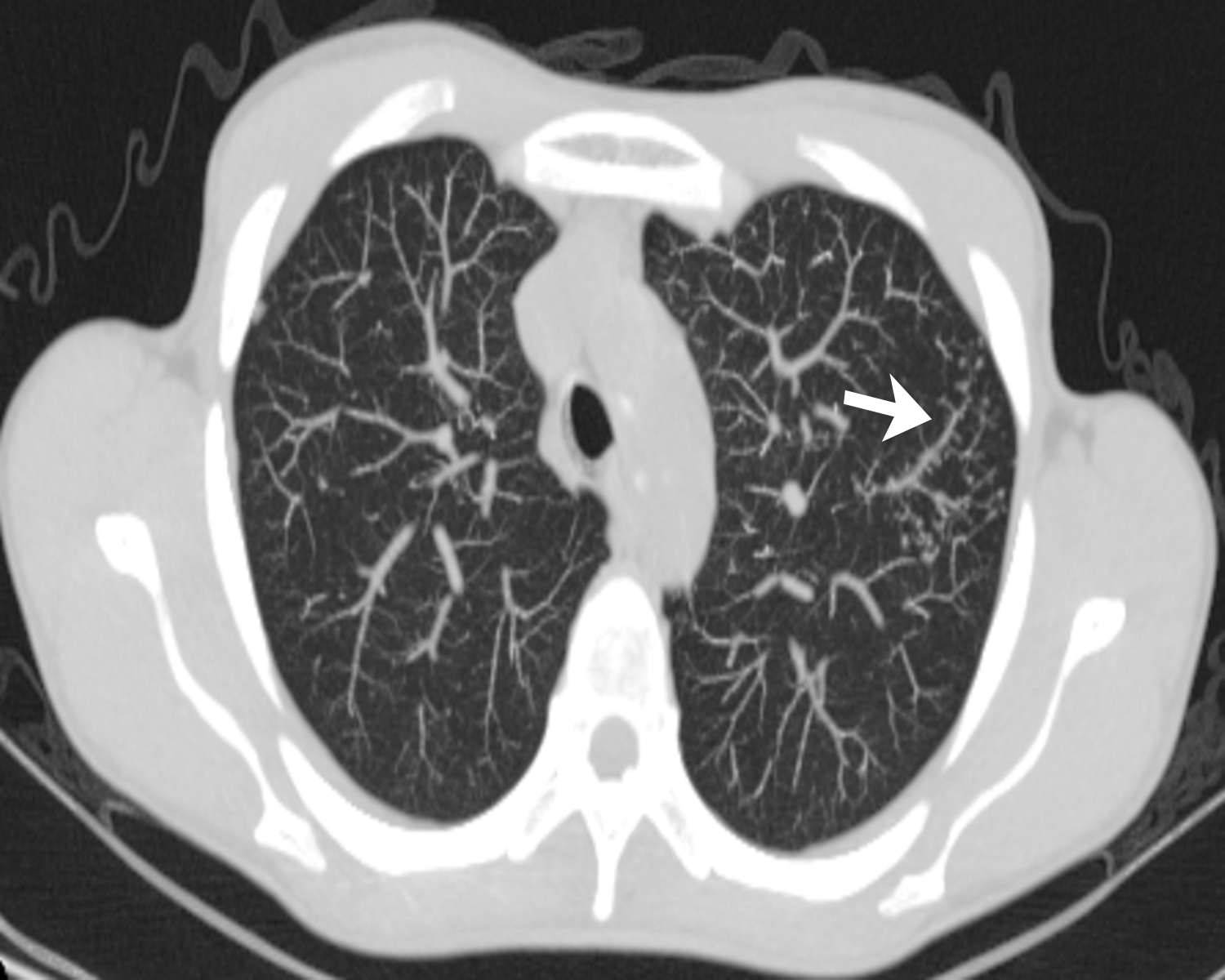

Supplement: S4 Fig — (TIF) [file pone.0186055.s004.tif]
