# Supplementary material for: CT Chest and pulmonary functional changes in patients with HTLV-associated myelopathy in the Eastern Brazilian Amazon
Source: PLoS One. 2017 Nov 2;12(11):e0186055. doi: 10.1371/journal.pone.0186055 (PMC5667869; doi:10.1371/journal.pone.0186055)
Supplement: S1 Table — (DOCX) [file pone.0186055.s005.docx]

**Abbreviation list**

**CT** computed tomography

**FVC** forced vital capacity

**FET_25%–75%_** 25%–75% forced expiratory time

**FEV_1_** forced expiratory volume in one second

**FEF_25–75%_** 25%–75% forced expiratory flow

**FEFmax** maximum forced expiratory flow

**FEF_50%_** 50% forced expiratory flow

**FEF_75%_** 75% forced expiratory flow

**HAM/TSP** HTLV-1-assoaciated myelopathy/tropical spastic paraparesis

**HTLV** Human T-lymphotropic virus type

**MVV** maximum voluntary ventilation

**OLD** obstructive lung disease

**RLD** restrictive lung disease

**VC** Vital capacity
